# General Anaesthesia With Multimodal Principles Versus Intrathecal Analgesia With Conventional Principles in Total Knee Arthroplasty: A Consecutive, Randomized Study

**DOI:** 10.4021/jocmr1210e

**Published:** 2013-01-11

**Authors:** Andreas Harsten, Hjortur Hjartarson, Mads Utke Werner, Soren Toksvig-Larsen

**Affiliations:** a Department of Anesthesiology, Hassleholm Hospital, 281 25 Hassleholm, Sweden; b Department of Orthopedic Surgery, Hassleholm Hospital, 281 25 Hassleholm, Sweden; c Multidisciplinary Pain Centre, Neuroscience Centre, Rikshospitalet, DK-2300 Copenhagen, Denmark

**Keywords:** General anaesthesia, Glucocorticoids, Intrathecal analgesia, Length of hospital stay, Postoperative recovery, Total knee arthroplasty, Urinary catheter

## Abstract

**Background:**

Postoperative outcomes following major surgery are influenced by surgical and anaesthesiological factors. While techniques of minimal invasive surgery have been associated with improved outcome, the techniques of minimal invasive, multimodal anaesthesia have not been adequately investigated. The aim of this study was to compare intrathecally based anaesthesia (ITA) including standardized, traditional intraoperative and postoperative care, with, general anaesthesia (GA) combined with intraoperative glucocorticoids, exclusion of intraoperative tourniquet and indwelling urethral catheter, and, an accelerated postoperative care regime. Outcome variables in the study were pain, requirement of analgesics, global satisfaction score and length-of-hospital stay.

**Methods:**

Sixty patients were included and randomized to the ITA or the GA group. The ITA group received intrathecal bupivacaine (12.5 - 15.0 mg)/morphine (0.1 mg)/clonidine (0.03 mg), a standard surgical procedure, local infiltration analgesia (LIA) with ropivacaine (110 mg) /epinephrine (0.5 mg)/morphine (10 mg), an indwelling urethral catheter and mobilization with start Day 1 after the surgery. The GA group received a target-controlled infusion of propofol/remifentanil, betamethasone 4 mg i.v. intraoperatively, surgery was performed without a tourniquet, an indwelling urethral catheter was not used, LIA was with ropivacaine (250 mg)/epinephrine (0.3 mg) and mobilization was planned with start ≤ 2 hrs. after end of surgery. Outcomes were followed daily for the first 96 hrs. and at visits 3 months and 12 months postoperatively.

**Results:**

Requirement of analgesics was decreased in the ITA group in the immediate postoperative period (P < 0.05). Pain scores were significantly lower in the ITA group (P < 0.01) between 0 - 12 hrs and in the GA group (P < 0.05) between 12 - 24 hrs after surgery. Fifteen of the patients in the GA group had to be intermittent catheterized due to bladder volumes > 400 mL. The LOS in the ITA group was significantly longer compared to the GA group (P < 0.01). There was no difference in global satisfaction score.

**Conclusion:**

General anaesthesia combined with intraoperative glucocorticoids and accelerated postoperative care, compared with, intrathecal blockade and traditional postoperative care, seems to generate the same overall pain ratings and a decrease in length-of-hospital stay, in patients undergoing elective total knee arthroplasty.

## Introduction

Total knee arthroplasty (TKA) is in most cases performed during general anesthesia (GA) or intrathecal analgesia (ITA) [1]. Despite a low rate of adverse effects and superior postoperative analgesia for ITA, there are disadvantages like failed segmental blockade, the necessity of an indwelling urethral catheter and potentially serious complications (hemorrhage, infection) [1, 2]. Furthermore, intrathecal analgesia with its profound motor block will often extend for several hours into the postoperative phase, compromising early mobilisation [1]. An effective alternative to an ITA-based regimen therefore, from a clinical pint of view, is highly desirable.

The primary aim of this study was to determine if GA combined with intraoperative glucocorticoids, exclusion of an intraoperative tourniquet, avoiding an indwelling urethral catheter and using an accelerated postoperative care regime, compared with ITA, with a standardized traditional intraoperative and postoperative care, could reduce postoperative pain ratings. Secondary outcome parameters included postoperative requirement of analgesics, global satisfaction score and length-of-hospital stay.

## Patients and Methods

The study was approved by the Research Ethics Committee at Lund University (no 144/20085). It was registered at www.clinicalTrials.gov (reg no NCT01604382). All patients gave their written informed consent prior to participating in the study.

### Study design

The study design was consecutive and randomized. Patients with osteoarthritis scheduled for TKA at the Department of Orthopedic Surgery, Hassleholm Hospital, Sweden, were eligible for participation in the study. Exclusion criteria were body mass index (BMI) > 35 m/kg^2^, prior major knee surgery to the ipsilateral knee, ongoing infection, known immunological deficiency or ASA physical status category ≥ IV.

### Randomization and blinding procedure

Randomization was performed by an employee, not involved in the study, who prepared non-transparent, sealed envelopes each containing a slip of paper with descriptions of whether the patient should receive GA or ITA. The randomization was computerized. On the study day a nurse, likewise not involved in the study, opened the appropriate envelope and prepared accordingly the procedures. From the point where anaesthesia was delivered both patients and staff in the operating theatre and in the recovery unit were, for obvious reasons, aware of the method of anaesthesia being used. However, once the patients left the recovery unit staff members assessing home readiness were blinded to group allocation.

### Assessments

All patients were familiarized with a horizontal visual analogue scale (VAS, (100 mm)) used for assessment of pain (0 = no symptom, 100 = worst symptom imaginable). Assessments of pain were made pre-operatively, upon arrival to the post-anaesthesia care unit (PACU), every second hour the first day, twice a day during the remaining hospital stay and at follow-up visits 3 and 12 months after surgery. Global satisfaction score was assessed with a VAS (0 = least possible satisfaction, 100 = best possible satisfaction) at follow-up visits 3 and 12 months after surgery.

### Anaesthesia

Oral premedication, administered 1 hour before surgery, was with midazolam 2.5 mg, paracetamol 2 g, meclizine 10 mg, celecoxib 200 mg and oxycodone 10 mg. A low-volume fluid regimen was used with 1,000 mL of Ringer’s solution (Fresenius-Kabi AB, 751 74 Uppsala, Stockholm) and 1,000 mL of glucose 2.5% during the first 24 hrs. Standard oral postoperative medication was with celecoxib 200 mg twice daily for 3 days, oxycodone 10 mg twice daily for 14 days and paracetamol 1 g four times daily for as long as the patients needed it. In the ITA group an indwelling urethral catheter was inserted prior to surgery. Intrathecal anaesthesia was with bupivacaine 12.5 - 15.0 mg, morphine 0.1 mg and clonidine 30 µg (total volume (3.0 - 3.6 mL). During surgery an infusion of propofol (0.8 - 2.5 mg/min) was used to induce a light level of sedation. At the end of surgery a mixture of ropivacaine 110 mg, epinephrine 0.5 mg and morphine 10 mg (total volume 21 mL) was injected as local infiltration analgesia (LIA) into the peri-surgical area. The mixture was injected using a systematic technique ensuring uniform delivery of the local anaesthetic to all tissues incised, handled or instrumented during the procedure. The first 7 mL of the mixture was injected into the posterior joint capsule and both collateral ligaments after the bone cuts had been performed. After insertion of the prosthesis another 7 mL was be injected along the borders of and into the capsule and the cut quadriceps tendon, infra-patellar ligament, possible remnants of the fat pad, cruciate ligaments and the soft tissues surrounding the joint. The last 7 mL was infiltrated into the subcutaneous tissues before wound closure [3].

In the GA group no urinary catheter was inserted. The patients were draped before induction of anaesthesia. Induction of anaesthesia was with propofol and remifentanil, and endotracheal intubation was facilitated by succinylcholine. Maintenance of anaesthesia was with target-controlled infusion (Marsh and Minto algorithm) with propofol (Braun Medical, Germany) and remifentanil (GlaxoSmithKline, Great Britain) aiming at initial concentrations of 5 µg/mL and 5 ng/mL, respectively [4, 5]. Ventilation was mechanical with oxygen in air (F_IO2_ = 0.45) and aimed at ETCO_2_ 4.5 kPa. Betamethasone (Swedish Orphan Biovitrum, Sweden) 4 mg i.v. was given during surgery. At the end of surgery a mixture of ropivacaine 250 mg and epinephrine 0.3 mg (100 mL) was injected in the tissues in the same way as described above. Twenty min before the end of anaesthesia an i.v. bolus dose of oxycodone 7.5 - 10 mg was given.

### Surgery

In the ITA-group a tourniquet was applied around the thigh before the start of surgery. The GA-group did not receive a tourniquet. The surgeries were performed via a ventral incision with a parapatellar medial entrance to the joint. The patella was everted. A cemented single radius cruciate retaining (CR) total knee was used (the Triathlon^TM^ Knee System (Stryker, Mahwah, New Jersey, USA)) for all patients. Appropriate guide instruments were used according to the surgical-technique manual supplied with the knee system.

### PACU

In the PACU intermittent doses of oxycodone 2 - 8 mg i.v. was given as rescue medication. Patients complaining of PONV were given ondansetron 4 mg i.v.

In the GA group bladder scans were done every second hour during the first 24 post-op hours, according to our routine clinical procedure. If the bladder contained > 400 mL in residual volume an intermittent catheterization was performed according to our routine clinical procedure. Mobilisation was started within 2 hrs. of arrival to the PACU. A physiotherapist did passive bending of the knee to 90° and walked 10 meters with the patient in the PACU.

In the ITA group mobilization was not started until the day after surgery, due to high prevalence of residual motor and sensory blockade.

### Ward

In the ITA group the indwelling urethral catheter was removed the day after surgery. Prior to removal a urine sample was taken for urine bacterial analyses. Patients were considered ready to be discharged from the hospital when [1] they were able to get in and out of bed [2], get dressed [3], sit down and get out of a chair [4], able to walk 50 m with or without walking aid [5], flex the knee to at least 70° [6], fit for staircase climbing under supervision of a physiotherapist and [7] accepting to be discharged. LOS was measured from end of surgery to time of discharge.

### Statistical analyses

Sample size was estimated using PS Power and Sample Size Program (http://biostat.mc.vanderbilt.edu/PowerSampleSize) and LOS was assessed the primary outcome. Data from previous randomized clinical trials indicated that 28 patients were needed in each group to demonstrate a 30% minimal relevant difference in pain score with a significance of 0.05 and a power of 0.80 [6]. To compensate for drop-outs, 30 patients were included in each group.

Data analyses were performed using SPSS version 17.0 (SPSS, Chicago, USA). Data-distribution was tested for normality with Shapiro-Wilks test and residual plots. According to data-distribution either Student-t test or Mann-Whitney U-test for un-paired data was used. Data are presented as mean (± SD) or median (25-75% interquartile range (IQR)). A P-value < 0.05 was assigned statistical significance.

## Results

**Figure 1:**
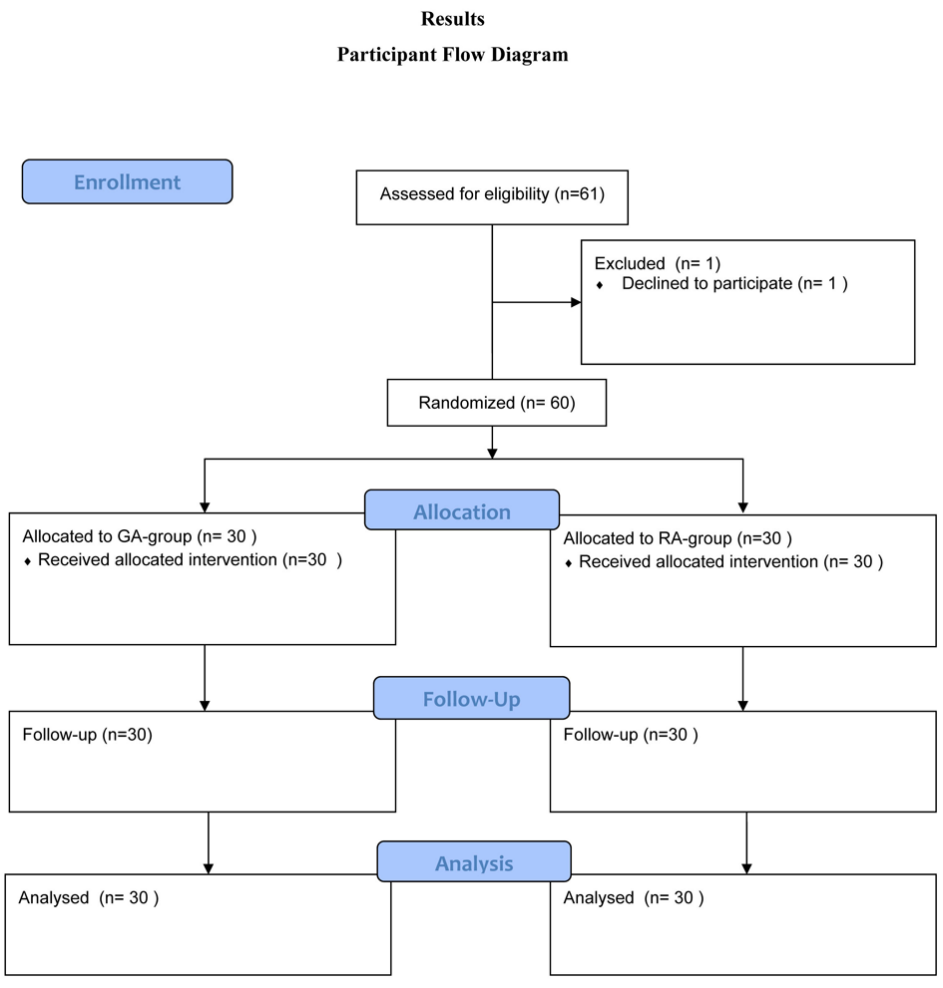
Consort Flow Diagram for the study.

Patients were recruited between September 2008 and May 2009. Sixty-one consecutive patients were assessed for eligibility by two orthopaedic surgeons and 60 patients were included after the pre-operative visit by the anaesthesiologist (Fig. 1) (CONSORT flow diagram). The 12 month follow-up was completed June 2010. Anthropometric and surgical data are summarized in Table 1.

**Table 1:** Demographics and Surgical Data

	GA-group ± SD	NA-group ± SD
	n = 30	n = 30
Weight (kg)	85.1 ± 12.7	82.2 ± 13.4
Height (cm)	173 ± 9	171 ± 10
Male/Female	16/14	16/14
Age (yrs)	67 ± 9	69 ± 7
ASA physical status		
I	11	10
II	16	18
III	3	2
Duration of surgery (min)	54 ± 11	60 ± 7
Per-operative bleeding (mL)	100 (50 - 150)	0 (0 - 50)

Weight, height age and duration of surgery presented as mean ± SD. Per -operative bleeding presented as median (IQR).

### Intraoperative variables

The estimated intraoperative blood loss was significantly higher in the GA group (P< 0.01, (Mann-Whitney test) (Table 1).

### Postoperative variables

**Figure 2:**
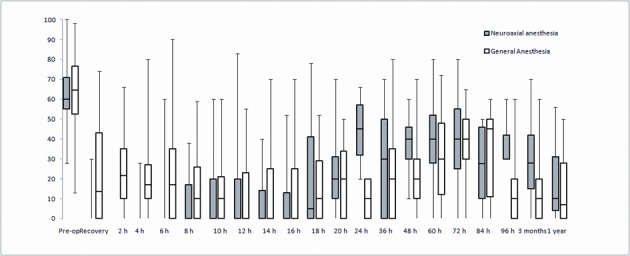
Pain scores for GA and NA groups. Line within the boxes indicate median and the boxes indicate 25-75% interquartile range (IQR). Whiskers indicate range. Area under the curve analyzed for 0 - 12, 12 - 24 and 24 - 48 hrs using Mann-Whitney test. Statistically significant differences (higher pain scores in RA group) between 0 - 12 hrs, *P* < 0.01 and (higher scores in NA group) between 12 - 24 hrs, *P* < 0.05.

Pain scores were significantly higher in the GA group between 0 - 12 hrs and in the ITA group between 24 - 48 hrs (Fig. 2) (Area under the curve analysed for 0 - 12, 12 - 24 and 24 - 48 hrs using Mann-Whitney test, P < 0.01 and P < 0.05).

In the first 24 hrs the requirements of oxycodone were 3 mg (IQR 2 - 5 mg) in the ITA group and 4 mg (IQR 2 - 7 mg) in the GA group (n.s. Mann-Whitney test). However the ITA group had a median time to first rescue pain medication of 605 (IQR 310 - 745) min as compared to 40 (IQR 9 - 68) min for the GA group until first rescue pain medication (P < 0.05 Mann-Whitney test).

In the GA group 15 of the 30 patients were intermittently re-catheterized at least once due to large bladder volumes. The bacterial analyses from urine samples in the ITA group were all negative. There were no differences in global satisfaction scores between the groups.

The length of hospital stay (LOS) was 80 (IQR 68 - 93) hrs in the GA group and 99 (IQR 84 -112) hrs in the NA group (P < 0.05, Mann-Whitney test).

## Discussion

Osteoarthritis of the knee is a debilitating chronic disease in the elderly [7]. In the United States alone, 550,000 TKAs were performed in 2007 [7]. To be able to perform such a large number of operations with a good surgical outcome in a health care system with huge economical restraints, clearly represents a formidable challenge. The aim of this study was to investigate whether a number of evidence-based changes from our standard procedure would improve post operative pain and reduce length of hospital stay without reducing patients’ global satisfaction.

### Brief summary of results

The patients in our study experienced maximum pain at different times during the post-operative period, but the summed up intensities of pain during 12 months after surgery did not differ between the groups. PONV was more frequent in the ITA group and the LOS was higher in the ITA group.

### Pain

We observed that the overall summed up intensities of pain and the requirement of rescue analgesic for 48 hrs after surgery did not differ between the groups. However, the GA patients experienced more pain in the early phase (0 - 12 hrs) of the postoperative period than the ITA patients, whereas the ITA patients experienced their maximum pain later (24 - 48 hrs) than the GA patients. The pain ratings observed in this study are comparable with previous findings [3]. Adding morphine and clonidine to intrathecal bupivacaine resulted in an opioid sparing effect and a decrease in pain intensity up 12 hrs [8, 9]. However, we monitored the pain scores for the first 4 postoperative days and again 3 and 12 months after surgery. At 3 months the patients in the GA group had a trend towards higher pain scores. Since the ITA group had less initial pain one might argue that this should reduce the risk for pain later on due to a decreased risk of development of central sensitization. Accordingly, the GA group that actually experienced pain early in the postoperative phase should have an increased risk for later pain problems due to central sensitization. This is speculative since the patients regardless of method of anaesthesia experienced equivalent pain.

It has convincingly been demonstrated that neuraxial analgesia results in less postoperative pain and morbidity, at least in high-risk patients, compared to general anaesthesia [10]. This study did not demonstrate overall differences between GA and ITA in terms of pain relief probably due to the use of glucocorticoids and accelerated recovery programme in the GA-based regimen [11, 12].

### Length-of-stay at hospital (LOS)

The LOS for TKA has substantially decreased during the last decade and is presently in the range of 3 - 5 days due to improvements in minimal invasive surgery and accelerated care principles [13]. The GA patients in our study stayed approximately 19 hrs. less in hospital compared to ITA patients. This difference may translate to an increase in cost-efficiency for the GA-regimen compared to the ITA-regimen.

### Tourniquet and bleeding

The use of tourniquet is associated with intraoperative, ischemic nociception [14]. During inflation of the tourniquet it is therefore important to maintain an adequate depth of anaesthesia. This increase in depth of anaesthesia may delay awakening and recovery and subsequently prolong the anaesthesia time. Furthermore, the use of a tourniquet makes it more difficult for the surgeon to detect lesioned blood vessels during the operation. In our study we found that avoiding tourniquet resulted in a median blood loss of 100 mL. This blood loss was statistically significant but hardly of any clinical significance. Interestingly, the blood loss was less and the duration of surgery shorter when compared to other studies [7].

### Urinary catheters

Patients in the GA group did not receive an indwelling urinary catheter, due to the minimal invasiveness of the regimen but 15/30 patients were intermittently re-catheterized. However, provided that bladder scans are done regularly during the first 24 postoperative hours, it might still be an advantage to avoid indwelling urinary catheters, since they are associated with a number of serious complications such as lower and upper urinary tract infections and subsequently deep wound infections [15, 16].

### Limitations

There are a number of limitations of the present study. Generally, these limitations are primarily incurred by the clinical important question: is one treatment regimen comparable or ever better than another treatment regimen, where both regimens consist of a number of adjustable treatment modalities. These problems have been debated for decades in the case of accelerated recovery programs, where standard randomization and blinding procedures have been considered extremely difficult to implement. Although such non-blinded studies may lack scientific rigor a number of these studies based on “best-evidence-available” have served as hypothesis-generating studies and attracted considerable clinical attention. Specifically, the confounding factors, are, first two different methods, intrathecal analgesia and general anaesthesia were compared without any postoperative blinding. Second, a number of different drugs and differing drug-doses, glucocorticoids, clonidine, epinephrine and morphine were used. Third, the postoperative rehabilitation measures differed between the groups and they were unblinded. These confounding factors obviously make it hard to single out the causality of single factors.

In conclusion this study shows that general anaesthesia, without tourniquet and without indwelling urinary catheter and with early mobilisation results in earlier discharge from hospital without adversely affecting pain or total satisfaction score.

## References

[1] Macfarlane AJ, Prasad GA, Chan VW, Brull R (2009). Does regional anesthesia improve outcome after total knee arthroplasty?. Clin Orthop Relat Res.

[2] McCartney CJ, McLeod GA (2011). Local infiltration analgesia for total knee arthroplasty. Br J Anaesth.

[3] Andersen LO, Husted H, Kristensen BB, Otte KS, Gaarn-Larsen L, Kehlet H (2010). Analgesic efficacy of intracapsular and intra-articular local anaesthesia for knee arthroplasty. Anaesthesia.

[4] Marsh BJ, Morton NS, White M, Kenny GN (1990). A computer controlled infusion of propofol for induction and maintenance of anaesthesia in children. Can J Anaesth.

[5] Minto CF, Schnider TW, Shafer SL (1997). Pharmacokinetics and pharmacodynamics of remifentanil. II. Model application. Anesthesiology.

[6] Andersen LO, Kristensen BB, Husted H, Otte KS, Kehlet H (2008). Local anesthetics after total knee arthroplasty: intraarticular or extraarticular administration? A randomized, double-blind, placebo-controlled study. Acta Orthop.

[7] Buvanendran A, Kroin JS, Della Valle CJ, Kari M, Moric M, Tuman KJ (2010). Perioperative oral pregabalin reduces chronic pain after total knee arthroplasty: a prospective, randomized, controlled trial. Anesth Analg.

[8] Popping DM, Elia N, Marret E, Wenk M, Tramer MR (2012). Opioids added to local anesthetics for single-shot intrathecal anesthesia in patients undergoing minor surgery: a meta-analysis of randomized trials. Pain.

[9] Engelman E, Marsala C (2013). Efficacy of adding clonidine to intrathecal morphine in acute postoperative pain: meta-analysis. Br J Anaesth.

[10] Liu SS (2012). Regional analgesia for postoperative pain: then & now. Anesth Analg.

[11] De Oliveira Jr., Almeida MD, Benzon HT, McCarthy RJ (2011). Perioperative single dose systemic dexamethasone for postoperative pain: a meta-analysis of randomized controlled trials. Anesthesiology.

[12] Romundstad L, Breivik H, Niemi G, Helle A, Stubhaug A (2004). Methylprednisolone intravenously 1 day after surgery has sustained analgesic and opioid-sparing effects. Acta Anaesthesiol Scand.

[13] Holm B, Kristensen MT, Myhrmann L, Husted H, Andersen LO, Kristensen B, Kehlet H (2010). The role of pain for early rehabilitation in fast track total knee arthroplasty. Disabil Rehabil.

[14] Estebe JP, Davies JM, Richebe P (2011). The pneumatic tourniquet: mechanical, ischaemia-reperfusion and systemic effects. Eur J Anaesthesiol.

[15] Balderi T, Carli F (2010). Urinary retention after total hip and knee arthroplasty. Minerva Anestesiol.

[16] Hameed A, Chinegwundoh F, Thwaini A (2010). Prevention of catheter-related urinary tract infections. Br J Hosp Med.

